# Foreign

**Published:** 1841-02-06

**Authors:** 


### FOREIGN.

*M. Andral on the value of Chemical and Physiological Experiments.*— . . . . . . . . . "The more complex that the composition of any animal fluid is, the more uncertain are the results of its analysis.

Take, for example, the blood: what do we really know about it? Little more, indeed, than that it is a fluid which holds in suspension numerous globules, of a determinate size, shape, and colour.

So difficult, indeed, is the chemical examination of organic substances, that we are continually finding chemists of the highest talents attributing to some of the fluids principles which are solely and altogether the result of their experiments in the laboratory. For example, Tiedeman and Gmelin, so celebrated for their analyses in organic chemistry, have admitted into the composition of the bile substances or principles which Dumas subsequently has most distinctly shown to be generated during the analytic operations to which they have been subjected.

Again, it is so difficult to reproduce phenomena altogether as nature has exhibited them, that often the substance which seems alike to our senses and our tests similar to another is, in reality, of a very different nature. Thus the (alleged) colouring matter of the blood, *hæmatosine*, separated by chemical means, does indeed exhibit characters which have led the most distinguished chemists to believe that it is to its presence that the peculiar colour of this vital fluid is owing;—and yet it does not redden on exposure to the air, or even to pure oxygen. It cannot, therefore, be quite the same substance as that which exists combined with the other elements of the blood . . . . . . We cannot, indeed, wonder much at the imperfection of organic chemistry, seeing that it is only by analysis that we can discover the nature or composition of organized substances; almost all attempts at the synthetic test having failed."

So much for the uncertainties of the results of chemistry applied to the discovery of organic bodies and of organic operations.

M. Andral proceeds next to point out the extreme difficulty of drawing accurate conclusions in physiology from experiments on living animals.

"In physiology," says he, "the difficulties increase; another element, which we have not to guard against in animal chemistry, arises here; the chemist has to do only with dead matter, whereas in this new department of natural science the body is still under the influence of the vital power. All the phenomena of a living animal body—at least among the higher classes of the scale—are so linked together, that you cannot touch one without making the others more or less suffer and sympathise. Do you try an experiment to detect a phenomenon which is carried on *au sein* of a living body? All the great functions are at once deranged in their mode of manifestation; more or less blood is lost; it no longer follows in the vessels its regular and normal course, and it becomes either quickened or retarded in consequence of pain and fright; the secretions are immediately altered; at the part where the incision is made, there is an afflux of the fluids and of sensibility; and that great vital property, excitability, is at once brought into play; in short, you induce a state of active hyperæmia, or inflammation.

Wherever you touch a living animal being, it is the nervous system which responds by disordered movements, and by alterations in the sensibility, and which thus at once disarranges the phenomena which you wish to produce. Hence the phenomenon, which you represent as isolated, is not in fact so; and for this reason, that no point can be touched without causing a greater or less amount of disturbance of the entire organism.

. . . . . . . . It is not, however, to be denied that, in certain cases, experimentation has thrown considerable light upon some points of physiology, and also of pathology. Thus, for example, the experiments on the fifth pair of cerebral nerves have afforded the point "de depart" of our knowledge of the morbid lesions of these nerves; we could not have known any thing on this subject, unless we had the data which experiments have supplied us with. It is by them also that the special functions of the portio dura have been discovered, and that certain pathological facts connected with these functions have been understood and explained. In other cases experiments on animals have been found to induce a train of morbid phenomena which are indisputably analogous with those which occur during certain diseases. Thus the injection of pus into the veins of animals has been observed to give rise to symptoms and appearances, which bear a striking resemblance with those exhibited in cases where a lesion of the blood has taken place spontaneously, as in scurvy, certain cases of typhus, the absorption of purulent matter, &c. Again, by experimenting we are enabled to study a certain number of the causes of diseases, as, for example, all the class of intoxications or poisonings—including under this term the introduction into the circulation not only of the common poisons from the mineral and vegetable kingdom, but also of miasms and of the various kinds of animal virus.

. . . . . . . . . . . . We find also, that by modifying the relative proportion of the constituents of the blood, various morbid states, which have their analogues in disease, may be induced. Thus, by withdrawing a certain quantity of the fibrine of the blood, we promote the effusion into various tissues; whereas by increasing its viscosity, a series of phenomena of an altogether different character is induced; as has been distinctly proved by the recent experiments of *Majendie.*

. . . . . . . . There is, therefore, a certain number of pathological lesions which may be explained by the results of direct experimentation.

We can excite inflammation and suppuration; but we cannot induce the formation of false membranes, of tubercles, of cancer, of hydatids, &c. Some specific cause or action is necessary to give rise to such morbid productions.

Assiduous attention, however, to various circumstances which are daily presented to our view, will probably enable us at length to advance in this obscure department of pathology. Already we know that the generation of hydatids in sheep is certainly promoted by feeding on wet pasturage." . . . . . .

With the following brief remarks on certain necroscopic fallacies, we shall close our extracts from M. Andral's lectures for the present.

. . . . . . . . . . "During hot weather, we not unfrequently observe on the lining membrane of the heart and great bloodvessels a well-marked redness, and the appearance of high vascular injection, which is the result not of an inflammatory action, as has been too often supposed, but altogether of the changes in the fluids and solids produced by incipient putrefaction. There are not a few pathological works which, I venture to say, are 'frappés de nullité,' from authors not being aware of this and such-like occurrences.

For example; it is generally admitted that, under the influence of malignant fever, the tissue of the spleen is usually more or less extensively altered. Now, the late M. *Bailly*, of Blois, whose premature loss to medical science every one must regret, has, it is well known, published some valuable works on malignant intermittent fever; and in these he has particularly dwelt upon the remarkable changes in the spleen; such, for example, as the soft diffluent disorganization of its tissue. But in all his observations no mention is ever made either of the lapse of time after death when the dissection took place, or of the heat of the weather at the time. Now, this very state of the spleen is found in every body, if the examination does not take place, during Summer, within thirty hours after decease. We cannot, therefore, rigorously adopt the statements of M. *Bailly*, as we are ignorant of the circumstances in almost every case which he has reported."—*Med. Chir. Rev. from Gazette des Medecins.*

### METEOROLOGICAL REGISTER FOR JANUARY, 1841.

*Kept at the Pennsylvania Hospital, by J. Conrad.*

| Date. | Thermometer. Max. | Thermometer. Min. | Thermometer. 9 A.M. | *Dew Point | Barometer. 10 A.M. | Barometer. 3 P.M. | Winds. | Rain. | REMARKS. |
|---|---|---|---|---|---|---|---|---|---|
| 1 | 32 | 23 | 24 | 18 | 30.04 | 29.52 | NE. | .750 | N. E. storm; sleet from 8 A. M. till 5 P.M.; low clouds from E. |
| 2 | 31 | 16 | 24 | 14 | 29.69 | 29.66 | SW.NW. | | Clear; blowing cold in evening. |
| 3 | 12 | 5 | 5 | —10 | 30.00 | 30.06 | NW. | | Cloudless; high wind. |
| 4 | 17 | 5 | 10 | 0 | 30.32 | 30.30 | W.SW. | | Partly clear; wind moderate. |
| 5 | 28 | 12 | 17 | 18 | 30.54 | 30.52 | NE. | | Cloudy; partly clear in aft.; rain in evening; wind brisk. |
| 6 | 53 | 28 | 44 | 43 | 30.34 | 30.26 | SE. | 1.156 | Rain all day; fog; calm. |
| 7 | 58 | 48 | 58 | 53 | 30.04 | 29.94 | S.SW. | .822 | Rain; wind high. |
| 8 | 50 | 37 | 44 | 36 | 30.13 | 30.08 | NW.N. | | Clear; evening partly clear; wind light. |
| 9 | 45 | 33 | 36 | 34 | 30.21 | 30.16 | NNW. | | Morning clear; afternoon and evening cloudy; wind light. |
| 10 | 39 | 35 | 36 | 34 | 30.19 | 30.13 | NE. | .055 | Light rain occasion'y; wind light. |
| 11 | 46 | 38 | 39 | 39 | 29.96 | 29.83 | NE.SW. | .392 | Rain in morn; fog all afternoon and evening; wind light. |
| 12 | 44 | 34 | 38 | 28 | 30.25 | 30.27 | NW. | | Clear; wind moderate; part clear in evening. |
| 13 | 36 | 34 | 36 | 33 | 30.21 | 30.05 | NE. | .531 | Snow from 7 to 8 AM; snow and sleet in aft.; rain in eve; w. mod. |
| 14 | 35 | 32 | 35 | 30 | 30.30 | 30.29 | NE. | .006 | Cloudy; wind brisk. |
| 15 | 36 | 32 | 33 | 32 | 30.30 | 30.28 | NE.N. | .143 | Drizzling in morn.; aft. and ev'g cloudy; lower clouds from NE. |
| 16 | 38 | 31 | 33 | 33 | 30.44 | 30.34 | NE.SE. | | Fog early in morning; cloudy all day. |
| 17 | 50 | 35 | 50 | 43 | 29.90 | 29.80 | SW.NW. | .812 | Rain in morn; afternoon cloudy; evening clear; wind moderate. |
| 18 | 34 | 12 | 19 | —3 | 30.45 | 30.52 | NW. | | Clear; hazy; wind high; thermometer 20° at noon. |
| 19 | 20 | 9 | 11 | —1 | 30.83 | 30.80 | NW. | | Morning hazy; afternoon and evening cloudy; wind light. |
| 20 | 32 | 20 | 22 | 23 | 30.66 | 30.51 | NE. | .700 | Snow storm all day, from 7 AM; sleet in evening. |
| 21 | 39 | 31 | 36 | 34 | 29.95 | 29.85 | NE.NW. | 2.053 | Heavy rain & high wind last n't; drizzling all day; sleet in ev'g. |
| 22 | 36 | 29 | 36 | 23 | 30.05 | 29.99 | NW. | | Cloudy. |
| 23 | 36 | 28 | 32 | 25 | 29.97 | 29.95 | NW. | | Cloudy. |
| 24 | 48 | 32 | 38 | 32 | 30.00 | 29.86 | SW.NW. | .002 | Morning cloudy; clear at noon; afternoon cloudy; ev'g clear. |
| 25 | 47 | 30 | 35 | 26 | 29.93 | 29.94 | SW.NW. | | Morning clear; afternoon cloudy; evening partly clear. |
| 26 | 40 | 28 | 33 | 25 | 30.34 | 30.29 | W.SW. | | Clear; evening partly clear; wind light. |
| 27 | 47 | 33 | 40 | 37 | 29.99 | 29.91 | SW. | .030 | Cloudy; rain from 5½ to 7 AM.; evening clear; wind light. |
| 28 | 47 | 33 | 40 | 32 | 30.20 | 30.22 | NW. | | Fair; hazy; wind light. |
| 29 | 39 | 34 | 37 | 35 | 30.21 | 30.00 | NE. | .385 | Rain and sleet from 8 AM. to 5 PM; wind brisk; clouds fm. E. |
| 30 | 43 | 33 | 37 | 27 | 30.14 | 30.10 | W. | | Cloudy; evening clear; wind moderate. |
| 31 | 44 | 28 | 36 | 27 | 30.16 | 30.08 | W.SW. | | Morn fair but hazy; afternoon cloudy; evening clear. |
| Mean | 38.77 | 27.75 | 32.71 | 26.5 | 30.18 | 30.11 | | 7.837 | |

| | | | |
|---|---|---|---|
| Mean temperature, | 33.26° | Clear days, - - | 11 |
| " pressure, | 30.14 inches. | Cloudy, - - | 11 |
| " dew-point, | 26.59° | Rain, snow, &c. - | 9 |

*Winds*—S. to W. 7 days; W. to N. 13 days; N. to E. 9 days; E. to S. 2 days.

* Average of three observations daily.

